# Beyond R-Genes: Dissecting Metabolic and Nutrient-Driven Wheat Rust Resistance Through Induced Mutagenesis

**DOI:** 10.3390/plants15142131

**Published:** 2026-07-10

**Authors:** Saule Kenzhebayeva, Alfia Abekova, Nargul Omirbekova, Sabina Shoinbekova, Saule Atabayeva, Gulina Doktyrbay, Aigul Amirova, Albrecht Serfling

**Affiliations:** 1 Biotechnology Department, Al-Farabi Kazakh National University, Al-Farabi Av. 71, Almaty 050041, Kazakhstan; nargul.tata@gmail.com (N.O.); shoinbekova@mail.ru (S.S.); sauleat@yandex.kz (S.A.); gulina_kaznu@mail.ru (G.D.); aigul_amir@mail.ru (A.A.); 2 Kazakh Research Institute of Agriculture and Crop Production, Erlepesov Street 1, Almalybak Village, Almaty Region 040909, Kazakhstan; aabekova@mail.ru; 3 Federal Research Centre for Cultivated Plants (JKI), Institute for Resistance Research and Stress Tolerance, Erwin-Baur-Str. 27, 06484 Quedlinburg, Germany; albrecht.serfling@julius-kuehn.de

**Keywords:** wheat, rust resistance, *Puccinia*, induced mutagenesis, APR, ROS, iron homeostasis, quantitative resistance

## Abstract

The increasing threat posed by wheat rust diseases caused by *Puccinia* spp. necessitates the development of resistance strategies that extend beyond conventional race-specific mechanisms. Although recent reviews (2023–2025) have emphasized gene discovery and genomic approaches, comparatively less attention has been given to the potential roles of metabolic regulation and micronutrient homeostasis in host–pathogen interactions. Here, we present a narrative synthesis of current evidence and propose a conceptual framework in which induced mutagenesis (ethyl methanesulfonate, EMS, and γ-irradiation) serves as a tool for investigating interactions among redox regulation, iron (Fe) homeostasis, and disease resistance. A key component of this framework is the proposed interplay between reactive oxygen species (ROS) signaling and Fe partitioning. Vacuolar iron transporters (VITs), ferritins, and associated transport networks regulate intracellular Fe distribution and may influence Fe availability at the host–pathogen interface, potentially affecting fungal development and host defense responses. This concept of “iron-withholding immunity” may operate alongside ROS-mediated defense processes, linking metabolism with immune function. Observations from mutant wheat populations are broadly consistent with the hypothesis that these processes may contribute to durable adult-plant resistance (APR), which is characterized by reduced disease development, coordinated defense responses, and relative stability across environments. In some studies, Fe-enriched mutant lines have been associated with enhanced expression of pathogenesis-related genes and the occurrence of combined APR and seedling-resistance phenotypes, suggesting possible links between micronutrient homeostasis and immunity. Integration of high-throughput phenotyping with genotype × environment × time (G × E × T) frameworks may further improve our understanding of quantitative resistance and disease-associated traits. Overall, this review highlights the potential importance of nutrient homeostasis, redox regulation, and susceptibility modulation as components of future research aimed at developing climate-resilient and nutritionally improved wheat cultivars.

## 1. Introduction

Wheat rust diseases caused by *Puccinia* spp. remain among the most destructive threats to global wheat production and grain quality. This enduring threat is largely driven by the pathogens’ rapid evolutionary dynamics and the frequent breakdown of deployed race-specific resistance genes [1,2,3,4,5,6,7,8]. Consequently, elucidating the biological mechanisms underlying durable rust resistance remains an important challenge in wheat pathobiology. While recent crop improvement efforts have primarily focused on resistance gene discovery and genomic selection, increasing evidence indicates that durable defense is governed by complex physiological, metabolic, and nutrient-regulated networks that extend far beyond classical gene-for-gene recognition [9,10].

Induced mutagenesis represents a powerful experimental and translational approach for generating novel genetic variation and investigating host defense mechanisms that may be difficult to identify within natural germplasm. Through chemical (e.g., ethyl methanesulfonate, EMS) and physical (e.g., γ-irradiation) treatments, mutagenesis generates diverse allelic perturbations affecting signaling pathways, redox regulation, nutrient transport, and transcriptional networks [11,12,13,14,15,16,17]. For example, induced single-nucleotide substitutions in resistance gene families have been shown to successfully convert susceptible phenotypes into complete resistance [12,17,18].

Beyond gene-specific effects, mutagenesis-derived populations provide valuable systems for investigating the broader metabolic reprogramming associated with host–pathogen interactions. Reactive oxygen species (ROS) signaling and micronutrient homeostasis, including Fe regulation, have been increasingly investigated as potential factors influencing fungal colonization and disease progression [19,20,21,22,23].

When integrated with high-throughput phenotyping platforms and genotype × environment × time (G × E × T) analytical frameworks, induced mutagenesis enables the dissection of spatio-temporal defense dynamics, including APR and slow-rusting phenotypes, by capturing genotype-dependent responses across environmental gradients and developmental stages [24,25,26]. Therefore, this review synthesizes current pathobiological insights derived from mutant wheat populations to develop a hypothesis-generating framework for investigating potential relationships among redox regulation, nutrient homeostasis, and durable rust resistance. By moving beyond exclusively gene-centric models, this perspective aims to highlight emerging research directions and identify opportunities for future investigation of physiological and metabolic contributions to wheat rust resistance.

## 2. Biology and Epidemiology of Wheat Rust Pathogens

Wheat rust diseases are caused by obligate biotrophic fungi of the genus *Puccinia*, which have co-evolved with their hosts and exhibit highly specialized infection strategies. The three major rusts—leaf rust (LR, *P. triticina*), stripe (yellow) rust (YR, *P. striiformis* f. sp. *tritici*), and stem rust (StR, *P. graminis* f. sp. *tritici*)—differ in host tissue preference, environmental optima, and epidemic behavior, yet all depend on living host cells for growth and reproduction [4,5,6].

Infection is initiated by airborne urediniospores that germinate on the leaf surface and penetrate host tissue primarily through stomata. Following entry, the fungus differentiates haustoria—specialized intracellular feeding structures surrounded by the host-derived extrahaustorial membrane—that serve as key interfaces for nutrient uptake and effector delivery. Through these structures, *Puccinia* spp. secrete effector proteins that suppress host immunity and reprogram cellular metabolism, enabling sustained colonization while maintaining host cell viability [27,28].

Epidemiologically, wheat rusts are characterized by high reproductive capacity, long-distance dispersal, and rapid population turnover [2,3,7,29,30]. Wind-borne urediniospores can spread across continents, facilitating the emergence and dissemination of new virulent races. Disease development is strongly influenced by environmental conditions, while the large population sizes and evolutionary potential of *Puccinia* spp. enable rapid adaptation to deployed host resistance genes [31,32,33,34,35]. Consequently, the identification of durable resistance mechanisms remains a major objective of wheat improvement programs.

## 3. Wheat–Rust Biotrophic Interface

The intimate interaction between *Puccinia* spp. and wheat represents a highly specialized biotrophic interface shaped by long-term co-evolution. Upon successful intercellular colonization, the fungus differentiates haustoria—specialized, invaginated feeding structures enveloped by the newly synthesized host extrahaustorial membrane. This complex interface functions as the interaction hub for both nutrient uptake and effector delivery [36,37,38,39,40].

Rust fungi deploy diverse effector proteins to manipulate host immunity and metabolism. In the apoplast, effectors suppress PTI by attenuating ROS bursts and masking fungal chitin via LysM-domain proteins, thereby evading recognition by host pattern recognition receptors (PRRs) [41,42]. Concurrently, cytoplasmic effectors are translocated across the extrahaustorial membrane into host cells, where they interfere with defense signaling pathways, inhibit PCD, and reprogram host metabolism to sustain biotrophic growth [43]. A subset of these effectors acts as avirulence (Avr) factors (e.g., AvrSr27, AvrSr35, and AvrSr50), which, if recognized by host intracellular nucleotide-binding leucine-rich repeat (NLR) receptors, trigger a robust effector-triggered immunity (ETI) [44,45,46,47].

In addition to their activity in the cytoplasm, some effectors are targeted to host organelles. For example, the chloroplast-targeted effector Pt48115 suppresses ROS accumulation and callose deposition, thereby weakening early physical and chemical defense responses [48]. Furthermore, nuclear-localized effectors directly reprogram host gene expression to favor pathogen accommodation and development [40,47,48,49].

Recognition of fungal Avr effectors by host intracellular nucleotide-binding leucine-rich repeat (NLR) receptors activates effector-triggered immunity (ETI), often culminating in a localized hypersensitive response (HR) that restricts biotrophic pathogen growth [45,46,47,48,49,50]. Collectively, the wheat–rust biotrophic interface represents a highly specialized environment in which nutrient acquisition, effector delivery, and immune recognition converge. The outcome of infection is determined by the dynamic interaction between pathogen effectors and host defense mechanisms operating at this interface. Understanding these processes provides a foundation for investigating how physiological and metabolic factors influence disease development and resistance.

## 4. Genetic Architecture of Seedling and Adult-Plant Resistance: A Metabolic Perspective

Seedling resistance (SR) and APR represent two distinct yet complementary layers of wheat defense against *Puccinia* spp. SR is typically mediated by single, race-specific NLR genes that recognize pathogen Avr effectors and trigger robust ETI, often culminating in a localized HR. While highly effective in the short term, this binary “all-or-nothing” defense exerts intense selective pressure on pathogen populations, frequently resulting in the rapid emergence of virulent races and the subsequent breakdown of deployed resistance [1,49].

In contrast, APR is quantitative, polygenic, and generally race-non-specific. It is governed by multiple loci that collectively modulate basal immunity, redox homeostasis, transport processes, and transcriptional regulation [2,35]. Rather than relying on direct effector recognition, APR mechanisms limit pathogen growth through coordinated metabolic adjustments, including the controlled accumulation of ROS, the reinforcement of cell walls, and the activation of pathogenesis-related genes [4,17]. Classic pleiotropic APR genes, such as *Lr34/Yr18/Sr57* and *Lr67/Yr46/Sr55*, encode transporter proteins that fundamentally alter the host’s physiological state—particularly sugar fluxes and membrane transport dynamics—thereby creating a restrictive apoplastic environment that hinders fungal colonization without inducing extensive host cell death [6,51,52].

At the population level, APR contributes to durable resistance by reducing pathogen reproductive output and slowing epidemic development, thereby lowering the evolutionary selection pressure for the emergence of novel virulent *Puccinia* races [1,53,54,55,56,57,58,59,60]. Consequently, modern breeding strategies increasingly emphasize “layered defense” architectures that combine major resistance genes with polygenic APR components. In this context, mutation-derived allelic diversity represents a valuable resource for exploring and optimizing complex defense networks. Such approaches may contribute to the development of climate-resilient wheat cultivars with improved agronomic, nutritional, and disease-resistance traits, although their long-term effectiveness will require validation across diverse genetic backgrounds and environmental conditions.

Micronutrient homeostasis, particularly Fe regulation, has been proposed as a potential contributor to quantitative resistance mechanisms and host–pathogen interactions [17,22,23]. Fe serves as an essential cofactor for numerous metabolic and antioxidant enzymes, while excess free Fe can promote ROS generation through Fenton-type reactions. Consequently, regulation of Fe distribution and ROS homeostasis may influence both host defense responses and pathogen development. However, the extent to which these processes directly contribute to APR in wheat remains incompletely understood. The emerging Fe–ROS concept warrants further experimental validation before its contribution to APR in wheat can be fully understood. Beyond Fe homeostasis, other nutrient-dependent processes also play important roles in determining the outcome of wheat–pathogen interactions. In particular, carbohydrate partitioning and sugar transport influence both host metabolism and pathogen nutrition. Biotrophic pathogens depend on a continuous supply of host-derived carbohydrates, and alterations in source–sink relationships can substantially affect pathogen growth, nutrient acquisition, and disease progression [10]. Increasing evidence suggests that some durable resistance mechanisms may operate through the modulation of nutrient fluxes and host physiology rather than direct pathogen recognition. Notably, the pleiotropic APR genes *Lr34* and *Lr67* encode transporter proteins rather than classical NLR receptors, supporting the concept that altered transport processes can contribute to quantitative disease resistance [51,52]. For example, the pleiotropic APR gene *Lr67* encodes a modified hexose transporter associated with reduced pathogen development and durable resistance to multiple rust diseases [51]. Subsequent functional studies have provided additional evidence that transporter-mediated resistance involves altered membrane transport processes and nutrient fluxes that may contribute to broad-spectrum disease resistance [61,62,63]. Likewise, changes in sugar transport, carbon allocation, and metabolic reprogramming can influence susceptibility by modifying nutrient availability at the host–pathogen interface [29]. Collectively, these observations indicate that nutrient-mediated immunity extends beyond Fe homeostasis and likely involves multiple interacting transport networks that regulate host defense, pathogen nutrition, and quantitative disease resistance. Consequently, the potential contribution of Fe homeostasis to disease resistance should be considered within the broader context of metabolic regulation and nutrient partitioning during wheat–pathogen interactions.

**Figure 1:**
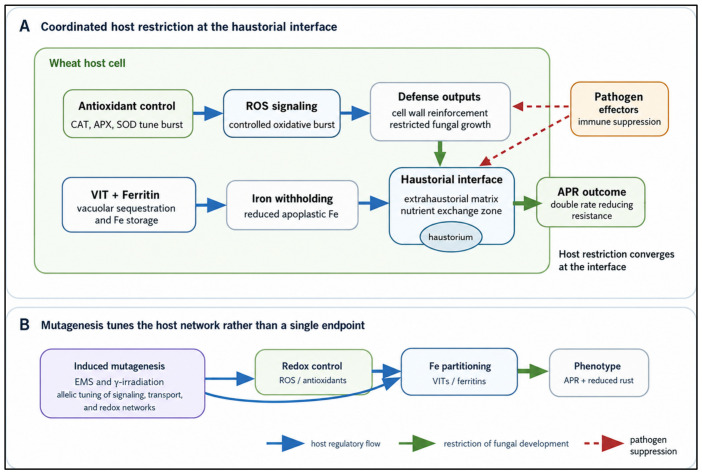
Hypothesized interactions between ROS signaling, Fe partitioning, and host defense responses during wheat–Puccinia interactions. (**A**) Proposed interactions between ROS signaling, Fe partitioning, and host defense responses during wheat–Puccinia interactions. Antioxidant systems, vacuolar iron transporters (VITs), ferritins, and pathogen effectors are shown in relation to the haustorial interface, where nutrient exchange and host–pathogen interactions occur. (**B**) Induced mutagenesis as a tool for generating variation in resistance-associated pathways. EMS and γ-irradiation produce allelic variation affecting redox regulation, transport processes, nutrient homeostasis, and disease-response phenotypes.

Studies of mutagenized wheat populations have demonstrated the utility of induced variation for identifying resistance-associated traits and generating novel combinations of SR and APR [11,12,13,14,15,16,17]. These populations provide valuable experimental resources for investigating the physiological, genetic, and metabolic processes that contribute to quantitative disease resistance. However, establishing causal relationships between specific mutations and resistance phenotypes requires further genetic, physiological, and multi-environment validation. More broadly, interactions between micronutrient homeostasis and disease resistance may provide opportunities to explore potential synergies between crop biofortification and pathogen defense. Similar links between Fe homeostasis, nutrient allocation, and immunity have been discussed in several plant systems [23,64], although direct evidence connecting biofortification-associated Fe accumulation with enhanced rust resistance in wheat remains limited. A conceptual illustration of these hypothesized interactions is presented in Figure 1.

## 5. Induced Mutagenesis as a Tool for Dissecting Durable Resistance Phenotypic Plasticity and Environmental Interactions

These approaches are particularly valuable because they enable the identification and characterization of physiological and metabolic processes that may contribute to quantitative resistance beyond classical race-specific immunity. Understanding the intricate layers of wheat immunity—from surface-level pattern-triggered immunity (PTI) to intracellular NLR resistosomes—provides the essential framework for applying functional genomics. By generating massive mutant populations, researchers can employ forward genetics to identify loss-of-function mutants that regain susceptibility, thereby validating the role of specific resistance loci. Conversely, reverse-genetic approaches can be used to investigate the contribution of signaling pathways, transcriptional regulators, transport processes, and stress-response mechanisms to disease resistance [63,64,65,66,67,68,69,70,71].

Chemical mutagens, particularly EMS, predominantly induce single-nucleotide substitutions by alkylating DNA bases. These point mutations are highly effective in modifying gene function in a subtle, non-lethal manner, which is critical for dissecting complex signaling components. This approach has enabled the disruption of specific kinase cascades [68,69], the identification of ‘master’ transcriptional regulators of the immune response (e.g., NAC and WRKY factors) [69], and the functional analysis of membrane transporters, such as the SWEET family [70]. Such variants clarify how altered transporter function, ROS homeostasis, and hormone-mediated signaling networks reshape quantitative resistance without invoking a fully race-specific, all-or-nothing response [66,67,68,69,70,71].

Physical mutagens, such as γ-irradiation, induce a broader spectrum of mutations, including large-scale deletions and chromosomal rearrangements [72]. These are particularly valuable for uncovering the polygenic, network-level effects associated with APR. For robust mutant generation, dose optimization is critical. For successful mutant generation, optimization of mutagen dose is critical and varies among wheat genotypes [16,21]. Combining physical mutagenesis with high-throughput phenotyping and sequencing-based genotyping facilitates the identification of genomic variation associated with disease-resistance phenotypes [16,21,73,74,75,76]. Combining this optimized physical mutagenesis with high-throughput phenotyping and sequencing-based genotyping facilitates a direct link between structural genomic variation and disease phenotype [16,21,73,74,75,76,77].

The transition from experimental mutant lines to commercial wheat cultivars is the next critical step in achieving durable rust resistance. Integrating induced mutagenesis into modern breeding programs offers strategic advantages, including accelerated pre-breeding via TILLING (Targeting Induced Local Lesions in Genomes) and speed breeding [78,79,80]. This combination enables researchers to swiftly identify and stabilize beneficial alleles within elite genetic backgrounds, drastically reducing the long breeding cycles traditionally associated with polygenic traits [16,81,82,83].

Despite its considerable utility, induced mutagenesis has several limitations that should be considered when interpreting resistance phenotypes. Mutations affecting transport processes, signaling pathways, or redox regulation may produce pleiotropic effects on plant growth, yield, nutrient accumulation, and abiotic stress responses [72,84]. Furthermore, mutagenized populations often carry substantial background mutation loads, complicating the identification of causal variants and underlying mechanisms [72]. The expression of APR is also strongly influenced by environmental conditions, developmental stage, pathogen population structure, and genetic background [1]. Consequently, resistance phenotypes observed in one environment may not be consistently expressed under different field conditions. These limitations highlight the importance of multi-environment validation, genetic characterization, and functional studies when translating mutation-derived discoveries into breeding applications.

Importantly, mutagenized populations provide experimental systems for investigating how alterations in signaling pathways, transport processes, nutrient homeostasis, and redox regulation influence quantitative resistance phenotypes. Consequently, induced mutagenesis serves not only as a breeding tool but also as a platform for testing hypotheses concerning the physiological and metabolic mechanisms discussed throughout this review.

**Figure 2:**
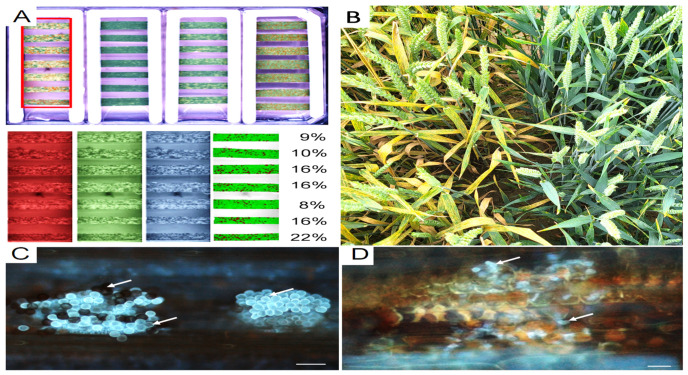
High-throughput phenotyping of non-mutated and mutated lines in a field trial, and microscopic evaluation of rust resistance in wheat mutant populations. (**A**) Automated differentiation of resistance responses in mutant lines was achieved using high-throughput digital image analysis of leaf-segment assays at the seedling stage. Multiple biological replicates were evaluated to quantify disease symptoms and classify resistance phenotypes. The red, green, and blue images represent the corresponding RGB channels extracted for image analysis, whereas the bright green binary images indicate the segmented disease lesions used for automated quantification. (**B**) Field evaluation of selected mutant lines was subsequently conducted at the adult plant stage under replicated trial conditions. Representative examples of a leaf and stripe rust susceptible non mutated (left) and a resistant mutant line (right) are shown. (**C**) Microscopic screening of the susceptible non mutated lines shows uredospore pustules seven days after leaf rust infection begins. The spherical uredospores are bright blue in colour. The colour turns dark brown 48 h after generation (bar = 80 µm). Spores within the pustule are marked by arrows. (**D**) Microscopic detection of degenerated pathogen structures (marked by arrows) and host responses after the start of infection. Defense-associated cellular responses, e.g., hypersensitive response and accumulation of phenolic compounds can be seen in yellow/orange coloured autofluorescence (bar = 20 µm). Spore pustules were not generated on the leaves of mutant lines.

The phenotypic expression of wheat rust resistance in mutant lines is inherently plastic, reflecting dynamic interactions among the host genotype, pathogen population structure, developmental stage, and environmental conditions (Figure 2) [8,16]. Mutation-derived resistance often manifests as quantitative reductions in disease severity, including decreased pustule density, delayed sporulation, and slower epidemic development (slow rusting), rather than complete immunity. Similar phenotypes have been reported for well-characterized APR genes such as *Lr34*, *Lr46*, and *Lr67*, which confer durable, quantitative resistance against multiple wheat rust pathogens [51,52]. Many quantitative resistance responses are developmentally regulated, becoming fully expressed only at later growth stages as host defense networks mature.

From a pathobiological perspective, understanding how induced mutations influence disease expression across diverse environmental conditions may help identify regulatory pathways associated with durable resistance. Importantly, mutagenized populations provide experimental systems for investigating how alterations in signaling pathways, transport processes, nutrient homeostasis, and redox regulation influence quantitative resistance phenotypes. Consequently, induced mutagenesis serves not only as a breeding tool but also as a platform for testing hypotheses concerning the physiological and metabolic mechanisms discussed throughout this review, including the potential interactions among nutrient partitioning, oxidative signaling, and disease resistance.

However, the relative contributions of genetic, physiological, and environmental factors remain incompletely understood and require further investigation across diverse genetic backgrounds and field environments [8].

## 6. High-Throughput Phenotyping to Quantify Host Responses to Rust Infection

High-throughput phenotyping (HTP) has become relevant to resolving quantitative host responses to rust infection in wheat, particularly in mutation-derived populations where resistance often manifests as subtle shifts in disease progression rather than complete immunity. Imaging-based approaches using visible light (RGB) are widely applied to quantify pustule density, lesion expansion, and overall canopy development, enabling the dynamic tracking of epidemic progression at a population scale (Figure 2) [16,75,85,86,87,88]. Furthermore, digital image analysis and hyperspectral imaging now enable the rapid and accurate evaluation of large breeding populations and mutant lines using high-throughput phenotyping approaches [85,87]. Beyond visible symptoms, complementary hyperspectral and chlorophyll fluorescence measurements can detect early physiological and photosynthetic changes associated with rust colonization [86,87,88]. Importantly, these spectral changes often precede visible pustule formation, enabling the detection of early physiological responses associated with pathogen colonization, including alterations in photosynthetic performance and other metabolic processes linked to defense activation [85,86,87]. In parallel, thermal imaging provides a rapid, non-destructive assessment of canopy temperature, which serves as an indirect indicator of stomatal conductance, transpiration, and plant water status. When integrated with HTP platforms such as unmanned aerial vehicles (UAVs) or autonomous gantry systems, thermal and hyperspectral imaging facilitate the multi-environment evaluation of disease development and mutation-derived resistance under field conditions [24,86,88]. Integrating these precise disease metrics with canopy structure and stress responses enables the identification of stable APR and other quantitative resistance phenotypes that are often difficult to detect reliably using conventional visual disease assessments [24,86,89]. Nevertheless, HTP-derived traits are often influenced by environmental conditions, sensor selection, and data-processing pipelines, highlighting the importance of independent validation and careful biological interpretation.

From a pathobiological perspective, HTP facilitates the high-resolution analysis of temporal defense responses, enabling researchers to discriminate between early pre-haustorial pathogen restriction, delayed disease development (slow-rusting), and stable APR. Repeated measurements across distinct developmental stages and environments reveal the complex genotype × environment × time G × E × T interactions that are important for interpreting the phenotypic plasticity and environmental responsiveness of mutant lines. Importantly, these approaches capture broader physiological consequences of infection—including altered transpiration, changes in photosynthetic performance, and premature senescence—thereby providing insight into the impact of disease on host physiology and plant performance [24,85,87]. Moreover, HTP is particularly valuable in mutation-derived populations because many resistance-associated phenotypes are quantitative, environmentally responsive, and difficult to detect using conventional visual assessments. By enabling the simultaneous measurement of disease progression, physiological responses, and stress-related traits across large populations, HTP provides a powerful platform for investigating the relationships among genetic variation, metabolic regulation, and disease resistance. Consequently, these approaches support the evaluation of the physiological and resistance-associated processes discussed throughout this review, including potential interactions among nutrient homeostasis, physiological responses, and durable resistance.

Integrating HTP with genomic, genetic, and mutation-derived datasets facilitates the identification of associations between induced genetic variation and quantitative disease traits. When combined with functional validation approaches, these datasets can help generate and test hypotheses regarding the mechanisms underlying disease resistance. Thus, HTP provides an important platform for connecting mutation-derived phenotypes with physiological and pathological responses, supporting the integration of phenomics, genetics, and pathobiology in studies of wheat rust resistance.

## 7. A Conceptual Fe–ROS–VIT Framework for Wheat Rust Resistance

Recent studies in plant biology and plant–microbe interactions have highlighted the importance of metabolic regulation in plant responses to biotic and abiotic stresses [90,91]. Among the processes receiving increasing attention are ROS signaling and Fe homeostasis, both of which influence plant physiology, stress responses, and host–pathogen interactions [92,93]. Emerging evidence suggests that interactions between these pathways may contribute to quantitative disease resistance, although the underlying mechanisms remain incompletely understood (Figure 2).

ROS are among the earliest defense responses activated upon pathogen recognition, contributing to defense signaling, cell wall reinforcement, and restriction of pathogen growth through the activation of downstream immune responses [92,93]. However, excessive ROS accumulation can also cause oxidative damage to host tissues and may impose physiological and metabolic costs on the plant if not properly regulated [92,93,94,95]. Iron plays a dual role in this process: while essential for plant metabolism, free Fe catalyzes hydroxyl radical production via the Fenton reaction, thereby modulating both immune responses and oxidative damage [96,97,98].

In wheat mutant lines, VIT-family transporters have been implicated in intracellular Fe partitioning and vacuolar sequestration and are associated with increased grain Fe accumulation and tissue-specific expression patterns related to Fe homeostasis [21]. These observations demonstrate the importance of VIT transporters in Fe homeostasis and biofortification. Whether similar transport processes influence Fe availability at the wheat–rust biotrophic interface remains an open question that requires further investigation. The concept of “iron-withholding immunity” suggests that host plants may restrict Fe accessibility to invading pathogens through the coordinated regulation of Fe transport, sequestration, storage, and redox-associated processes [23,98]. In several plant–microbe systems, such mechanisms have been associated with reduced nutrient availability for pathogens and altered disease outcomes [23,98]. However, direct evidence that VIT-mediated Fe sequestration contributes to reduced haustorial function or durable rust resistance in wheat remains limited. Likewise, Fe compartmentalization may contribute to the regulation of ROS homeostasis and defense responses, although the mechanistic relationships among Fe partitioning, redox regulation, and rust resistance require further functional validation. At the haustorial interface, *Puccinia* spp. depend on host-derived nutrients to sustain colonization. The extent to which Fe availability contributes directly to this process in wheat rust pathosystems remains incompletely understood. Host plants regulate intracellular Fe distribution through coordinated networks of transporters, storage proteins, and metal chelators, including vacuolar iron transporters (VITs) and ferritins [23,96,97,98]. It has been proposed that these processes may influence Fe availability at the host–pathogen interface and thereby affect pathogen development. This concept, often referred to as “iron-withholding immunity”, is supported by studies in several plant–microbe systems, although direct evidence in wheat rust pathosystems remains limited [23,98]. Furthermore, interactions between Fe homeostasis and ROS regulation may influence defense responses and oxidative stress management during infection [92,93,96,97].

Observations from resistant and mutagenesis-derived wheat genotypes are broadly consistent with this conceptual framework. Several studies have reported coordinated changes in antioxidant enzyme activity, redox homeostasis, and pathogen proliferation during wheat rust infection [86,94,95]. In some resistant genotypes, reduced pathogen growth has been associated with enhanced antioxidant responses, including increased activities of catalase (CAT) and ascorbate peroxidase (APX), together with broader metabolic reprogramming [86,94,95]. These observations suggest potential links among redox regulation, host metabolism, and disease resistance. However, the causal relationships among Fe homeostasis, ROS signaling, and rust resistance remain incompletely resolved, and the available evidence does not yet establish direct mechanistic connections among these processes [2].

Recent advances in wheat biofortification research emphasize the importance of Fe transport, partitioning, and storage for grain nutritional quality [17,99]. These findings raise the possibility that components of Fe homeostasis may influence both nutritional traits and disease responses. By linking Fe homeostasis with immune signaling and redox regulation, this conceptual model provides a basis for exploring potential connections between micronutrient biofortification and durable disease resistance (Figure 2). However, direct evidence linking biofortification-associated Fe accumulation with enhanced rust resistance remains limited.

It should be emphasized that this conceptual model is intended to integrate emerging observations from studies of Fe homeostasis, ROS signaling, and quantitative disease resistance. While individual components are supported by experimental evidence, direct causal relationships among all components have not yet been established in wheat rust pathosystems. Future research integrating functional genetics, transporter biology, pathogen infection assays, and multi-environment phenotyping will be required to rigorously evaluate these hypotheses and clarify the contribution of Fe homeostasis to durable rust resistance in wheat under diverse environmental conditions.

## 8. Genomic Dissection and Systems Integration of Mutation-Derived Resistance

The genomic and breeding approaches discussed below are particularly relevant because they provide tools for identifying and validating candidate genes and pathways associated with quantitative resistance, including transport processes, redox regulation, nutrient homeostasis, and other physiological mechanisms discussed throughout this review. Genomic and transcriptomic analyses have contributed substantially to our understanding of how mutation-derived variation may influence wheat rust resistance at both gene-specific and systems levels. By linking induced genetic variation to altered disease phenotypes, high-resolution genotyping and gene expression profiling provide an important basis for identifying candidate resistance mechanisms and regulatory pathways [100,101]. In EMS-mutagenized populations, where point mutations predominate, genotyping approaches such as exome capture, targeted amplicon sequencing, and whole-genome resequencing are indispensable for functionally interpreting induced variation within the large and complex hexaploid wheat genome [11,101,102]. While exome capture and targeted sequencing enable the efficient detection of allelic variation in coding and regulatory regions, whole-genome resequencing provides higher-resolution characterization of structural variation and background mutation load, thereby facilitating the identification of candidate loci and pathways associated with rust resistance [11,101,102,103].Conversely, the genotyping of γ-irradiation-derived mutants emphasizes the detection of broader structural variations, including large deletions and copy number changes, utilizing resequencing-based read-depth analysis, long-read sequencing, and modern cytogenetics. Although fine mapping in mutagenized populations can be challenging because of background mutations and, in some populations, structural genomic alterations, these approaches can reveal coordinated changes affecting multiple defense-related pathways and regulatory networks that may be associated with disease-resistance phenotypes under field conditions [11,13,102]. Functional validation integrates high-resolution genotyping with HTP and complementary molecular approaches to investigate associations between induced variants and disease outcomes. When integrated with reverse genetics, transcriptomic analyses, and functional validation, these approaches can help identify candidate genes and generate mechanistic hypotheses regarding disease resistance [104,105,106]. Crucially, mutation-derived alleles are increasingly evaluated across multi-environment trials to assess the robustness and stability of these resistance mechanisms. Together, integrated genotyping and functional validation provide a rigorous framework for distinguishing core defense regulators from environment-dependent modifiers.

Once identified and functionally validated, these resistance alleles and regulatory networks can be deployed in elite breeding germplasm through marker-assisted selection (MAS) for major-effect loci, genomic selection (GS) for polygenic resistance, and genome editing (GE) for targeted improvement of disease-resistance traits [107,108,109,110,111]. Rather than replacing classical biological discovery, these approaches serve as translational tools that operationalize the knowledge of defense pathways and genotype × environment interactions revealed by mutation-derived analyses.

MAS remains an important tool for tracking favorable alleles in segregating populations, particularly for resistance traits governed by major genes or discrete quantitative trait loci (QTL), while complementary genomic approaches increasingly support the dissection of more complex resistance architectures [107,108,111]. Mutation-derived alleles often modulate core components of basal immunity, transport, and transcriptional regulation, making them particularly suitable for MAS once causal variants or tightly linked markers have been established. Unlike race-specific resistance genes, these alleles typically contribute to quantitative APR, generally reduce the selection pressure imposed on pathogen populations and thereby contribute to more durable resistance [8,51,52]. Furthermore, genomic selection (GS) enables the integration of mutation-derived resistance by capturing the cumulative effects of multiple small-effect loci that shape defense robustness across environments. GS models, particularly when informed by high-throughput phenotyping, can predict the performance of resistance traits under diverse epidemiological conditions, thereby aligning selection decisions with the biological complexity of host–pathogen interactions [35,108,110].

Finally, genome editing (GE) provides a promising means of translating insights from functional genomics into crop improvement by enabling the precise modification and functional validation of defense-associated genes [108,109]. Functional characterization of induced alleles can identify candidate genes suitable for targeted editing, including regulators of immune signaling, transport processes, and stress responses [102,108]. Targeted modification of susceptibility (S) genes has emerged as a particularly promising strategy because it may reduce the dependence of resistance on specific pathogen-recognition events [109]. Emerging studies also suggest that genes involved in nutrient transport and Fe homeostasis, including VIT-family transporters, may represent potential targets for future investigation. However, the extent to which these pathways contribute directly to durable rust resistance remains incompletely understood, and further functional validation will be required before their deployment in resistance-breeding programs.

Despite these advances, translating candidate resistance loci into robust field performance remains challenging because disease expression is influenced by complex interactions among genotype, environment, developmental stage, and pathogen variability [7,110,112]. Consequently, validation across diverse environments, pathogen populations, and genetic backgrounds remains essential for assessing the durability and practical value of candidate resistance mechanisms [108,112].

## 9. Future Perspectives: Mutagenesis as a Systems Tool for Wheat Rust Pathobiology

Future progress in wheat rust pathobiology will increasingly rely on approaches that integrate targeted genetic perturbation with systems-level biological interpretation [12]. In addition to generating novel resistance phenotypes, induced mutagenesis introduces genetic variation affecting signaling pathways, regulatory networks, and metabolic processes involved in wheat–rust interactions [12,13,16,17]. Well-characterized mutant populations therefore provide valuable resources for investigating resistance mechanisms across developmental stages and environmental contexts [13,16,17].

Moving forward, integrating mutagenesis with high-throughput phenotyping (HTP) and multi-omics analyses—including transcriptomics, proteomics, and metabolomics—has considerable potential to improve our understanding of how quantitative resistance emerges from interactions among immune signaling, metabolism, and host physiology [12,113,114]. As multi-scale datasets continue to expand, machine learning and network-based approaches may help identify candidate regulatory pathways and generate predictive models of disease responses under diverse environmental conditions [108,113].

Furthermore, future research should prioritize evaluating mutation-derived resistance-associated networks under combined biotic and abiotic stresses. As climate change increasingly exposes wheat to concurrent challenges, such as rust infection and drought stress, it will be important to determine how pathways associated with Fe homeostasis, ROS regulation, nutrient transport, and stress adaptation interact under multi-stress conditions. In this context, interactions among micronutrient homeostasis, oxidative signaling, and disease resistance represent promising areas for future investigation. Ultimately, these approaches may contribute to a more comprehensive understanding of host–pathogen interactions and facilitate the development of improved strategies for enhancing durable resistance under changing environmental conditions. However, continued functional validation will be required to distinguish causal mechanisms from correlative associations and to assess the stability of resistance across diverse genetic backgrounds and environments. Future advances will depend not only on the identification of candidate resistance-associated pathways but also on rigorous experimental validation of their biological functions and contributions to disease resistance under field-relevant conditions.

## 10. Conclusions

The continuing threat posed by wheat rust pathogens highlights the need for a comprehensive understanding of host defense mechanisms operating across multiple biological scales, from immune signaling and pathogen recognition to physiological regulation, nutrient homeostasis, and environmental adaptation. While natural genetic diversity remains the foundation of resistance breeding, induced mutagenesis provides a valuable experimental platform for generating novel variation and investigating the complex processes associated with durable APR.

Chemical mutagens (e.g., EMS) and physical mutagens (e.g., γ-irradiation) generate complementary forms of genetic variation that facilitate the functional analysis of resistance-associated traits. When integrated with HTP, genomic approaches, and multienvironment (G × E × T) evaluations, these resources enhance our ability to identify and characterize quantitative resistance phenotypes, including slow-rusting responses and environmentally responsive APR mechanisms.

This review highlights emerging evidence that interactions among redox regulation, micronutrient homeostasis, and host metabolism may contribute to wheat rust resistance. In particular, interactions among Fe partitioning, ROS signaling, nutrient transport, and disease resistance warrant further investigation as potential contributors to quantitative resistance mechanisms. However, direct mechanistic evidence connecting all components of this framework remains limited, and further functional validation will be required to establish causal relationships in wheat rust pathosystems.

Looking forward, the integration of induced mutagenesis, systems biology, GE, and advanced phenotyping offers promising opportunities to investigate resistance-associated pathways and accelerate crop improvement. Continued validation across diverse genetic backgrounds, environmental conditions, and pathogen populations will be essential to determine the stability, biological significance, and breeding value of mutation-derived resistance mechanisms. Such efforts may ultimately contribute to the development of wheat cultivars with improved disease resilience, agronomic performance, and nutritional quality under increasingly challenging agricultural conditions.

## Data Availability

No new data were created or analyzed in this study.

## Abbreviations

The following abbreviations are used in this manuscript:

APR	Adult-plant resistance, quantitative
Avr	Avirulence
GE	Gene editing
G × E × T	Genotype × environment × time
GS	Genomic selection
HRG	High-resolution genotyping
HTP	High-throughput phenotyping
HR	Hypersensitive response
EMS	Ethyl methanesulfonate
ETI	Effector-triggered immunity
LR	Leaf rust
NLR	Nucleotide-binding leucine-rich repeat
MAS	Marker-assisted selection
MASA	Marker-assisted segregation analyses
PTI	Pattern-triggered Immunity
PRRs	Pattern recognition receptors
PCD	Programmed cell death
ROS	Reactive oxygen species
RR	Rust resistance
SR	Seedling resistance
StR	Stem rust
S	Susceptibility genes
VIT	Vacuolar iron transporters
YR	Stripe (yellow) rust
